# The prevalence and health consequences of frailty in a population-based older home care cohort: a comparison of different measures

**DOI:** 10.1186/s12877-016-0309-z

**Published:** 2016-07-07

**Authors:** Michael A. Campitelli, Susan E. Bronskill, David B. Hogan, Christina Diong, Joseph E. Amuah, Sudeep Gill, Dallas Seitz, Kednapa Thavorn, Walter P. Wodchis, Colleen J. Maxwell

**Affiliations:** Institute for Clinical Evaluative Sciences, 2075 Bayview Ave., Toronto, ON M4N 3M5 Canada; Division of Geriatric Medicine, University of Calgary, HSC-3330 Hospital Drive NW, Calgary, AB T2N 4N1 Canada; School of Epidemiology, Public Health & Preventive Medicine, University of Ottawa, 451 Smyth Road, Ottawa, ON K1H 8M5 Canada; Department of Medicine, Queen’s University and St Mary’s of the Lake Hospital, 340 Union Street, Kingston, ON K7L 5A2 Canada; Division of Geriatric Psychiatry, Queen’s University and Providence Care, 752 King Street W., Kingston, ON K7L 4X3 Canada; Ottawa Hospital Research Institute, 501 Smyth Road, PO Box201B, Ottawa, ON K1H 8L6 Canada; Institute of Health Policy Management & Evaluation, University of Toronto, 155 College Street, Toronto, ON M5T 3M6 Canada; Schools of Pharmacy and Public Health & Health Systems, University of Waterloo, 200 University Ave. W., Waterloo, ON N2L 3G1 Canada

**Keywords:** Frailty, Home care, Older adults, Health outcomes, Predictive validity

## Abstract

**Background:**

Evaluating different approaches to identifying frail home care clients at heightened risk for adverse health outcomes is an important but understudied area. Our objectives were to determine the prevalence and correlates of frailty (as operationally defined by three measures) in a home care cohort, the agreement between these measures, and their predictive validity for several outcomes assessed over one year.

**Methods:**

We conducted a retrospective cohort study with linked population-based administrative and clinical (Resident Assessment Instrument [RAI]) data for all long-stay home care clients (aged 66+) assessed between April 2010–2013 in Ontario, Canada (*n* = 234,552). We examined two versions of a frailty index (*FI*), a *full* and *modified FI*, and the *CHESS* scale, compared their baseline characteristics and their predictive accuracy (by calculating the area under the ROC curve [AUC]) for death, long-term care (LTC) admission, and hospitalization endpoints in models adjusted for age, sex and comorbidity.

**Results:**

Frailty prevalence varied by measure (19.5, 24.4 and 44.1 %, for *full FI*, *modified FI* and *CHESS,* respectively) and was similar among female and male clients. All three measures were associated with a significantly increased risk of death, LTC admission and hospitalization endpoints in adjusted analyses but their addition to base models resulted in modest improvement for most AUC estimates. There were significant differences between measures in predictive accuracy, with the *full FI* demonstrating a higher AUC for LTC admission and *CHESS* a higher AUC for hospitalization - although none of the measures performed well for the hospitalization endpoints.

**Conclusions:**

The different approaches to detecting vulnerability resulted in different estimates of frailty prevalence among home care clients in Ontario. Although all three measures were significant predictors of the health outcomes examined, the gains in predictive accuracy were often modest with the exception of the *full FI* in predicting LTC admission. Our findings provide some support for the clinical utility of a comprehensive *FI* measure and also illustrate that it is feasible to derive such a measure at the population level using routinely collected data. This may facilitate further research on frailty in this setting, including the development and evaluation of interventions for frailty.

**Electronic supplementary material:**

The online version of this article (doi:10.1186/s12877-016-0309-z) contains supplementary material, which is available to authorized users.

## Background

In Canada, formal or publically provided home care has been identified as an important component of the health care system [1]. In 2012, nearly 2.2 million Canadians received help or care at home because of a long-term health condition, disability, or problems related to aging [2]. The provision of formal home care services has the potential to significantly reduce utilization of acute care services and delay long-term care (LTC) admission [3–5]. An important public health priority is evaluating possible approaches to identifying those clients most at-risk of adverse health outcomes [6–8] to facilitate targeted interventions within this population [9, 10].

The concept of frailty offers a promising avenue for identifying and targeting care to home care clients at risk of a decline in their health and/or functional status [9–11]. Frailty is a state of heightened vulnerability to stressors due to cumulative decline across multiple physiological systems [11, 12]. Although frailty research on home care clients is relatively scarce, community-based studies indicate that the prevalence of frailty increases with advancing age (to over 30 % among those aged 85 and older) and is more common among women [12–16]. Frailty is also a significant predictor of disability, hospitalization, and mortality independent of measures of comorbidity [12–14, 17].

A number of approaches to screen for frailty and/or grade it have been proposed with no consensus on which one to use [18–20]. One of the most commonly used approaches involves the calculation of a *Frailty Index (FI)* based on a count of accumulated health deficits (i.e., symptoms, signs, diseases, disabilities, and/or laboratory abnormalities) divided by the number of potential deficits considered in a given individual [21]. In deriving a *FI* measure, typically 30 or more health “deficits” are selected that meet the following criteria: (i) they are associated with health status; (ii) cover a range of systems/domains (e.g., physical, cognitive, psychosocial); and (iii) increase with age but do not saturate (become universal) at older ages. Demonstrating the feasibility of deriving a frailty measure like the *FI* from routinely collected data, as well as its clinical utility, is appealing given the potential to inform research, care and health system planning for home care clients at a population level.

*FI measures* have been calculated for older adults receiving care in both assisted living [22, 23] and home care settings [24] using items derived from a standardized, comprehensive clinical assessment, the Resident Assessment Instrument (RAI). As might be expected given the flexibility in selecting items for a *FI*, there was variation across these studies in the number and range of the specific deficits or items included [22, 24]. Another approach to the detection of frailty based on RAI data is the *Changes in Health, End-stage disease and Signs and Symptoms* (*CHESS*) scale [25, 26]. Higher scores on the *CHESS* scale have been shown to predict mortality and hospitalization among home care recipients, as well as residents of assisted living and LTC facilities [22, 24, 25, 27]. Although these different approaches to identifying frailty might reasonably be expected to capture distinct subgroups of vulnerable clients, with varying implications for clinical or social care and interventions, few studies have directly compared different measures in terms of their descriptive characteristics or predictive validity for major health outcomes in older adults.

In our previous assisted living study [22], we showed that two *FI* measures (varying in item number and domains covered) and the *CHESS* scale modestly improved the performance of predictive models for mortality and LTC placement. The more comprehensive *FI*, comprised of 83 items covering a diverse range of domains, performed significantly better than the other approaches in predicting institutionalization [22]. Using RAI data available for a large sample of older home care clients in Ontario, Armstrong et al. [24] found that higher frailty levels as defined by three measures, including the *CHESS* scale and a 50 item *FI* were associated with a greater risk of a composite adverse outcome (i.e., death or institutionalization). Although informative, both studies present limitations in generalizing to a broader home care population. With our previous study, it is important to note the differences between residents of assisted living facilities and home care clients in sociodemographic and functional characteristics and in their care settings, staff and service availability [22–24]. The study by Armstrong et al., [24] evaluated clients from select catchment areas only, employed a less comprehensive *FI* measure, and limited their analysis to a single composite outcome.

To address these limitations, we utilized population-based linked administrative health and RAI data for a cohort of older home care clients in Ontario, to: (i) determine the prevalence and correlates of frailty in a home care population as operationally defined by two versions of a *FI* and *CHESS* scale; (ii) examine the agreement between these measures; and, (iii) compare the relative associations of these three measures (and their predictive validity) with death, institutionalization, and hospitalization (including alternate level of care bed stay) during one year of follow-up.

## Methods

### Study design, setting, and population

We conducted a retrospective, population-based cohort study of long-stay home care clients by linking Resident Assessment Instrument for Home Care (RAI-HC) data with multiple health administrative datasets from Ontario, Canada. These datasets were linked using unique encoded identifiers and analyzed at the Institute for Clinical Evaluative Sciences.

Ontario has a diverse, multicultural population of approximately 13 million people with nearly all residents eligible for medically necessary physician and hospital services in the publicly funded health care system. In addition, a variety of community and mental health services, a portion of the cost of care in LTC facilities, and most of the cost of prescription drugs for individuals aged 65 years and older, people on social assistance, and Ontario residents facing high drug costs are also provided through the publicly funded system. Access to home care services is centrally managed through 14 regional coordination centres (Community Care Access Centres) across the province [28]. Services are provided on a short- or long-stay basis with the latter referring to clients receiving ongoing supportive care for more than 60 days in a single episode. In Ontario, the RAI-HC assessment is only mandatory for all long-stay clients [6, 7, 29].

The RAI-HC and the home care service use databases were used to identify all long-stay home care clients assessed between April 2010 and March 2013. The RAI-HC is administered by trained professionals and provides a standardized assessment of clients’ physical and cognitive status, health conditions, behavioural problems, medications, and service utilization [30, 31]. Previous work has shown support for the reliability and validity of the RAI home care tool [31]. For individuals with multiple assessments, we utilized information from their earliest assessment during the study period (*index date*). We excluded clients aged <66 years or >105 years, those receiving home care for case management purposes only, individuals with a prior LTC stay, individuals who died on or prior to cohort entry, and clients with a non-Ontario postal code.

Ethics approval was obtained from the University of Waterloo Research Ethics Committee and the Research Ethics Board of Sunnybrook Health Sciences Centre, Toronto, Ontario, Canada.

### Frailty measures

Three separate measures of frailty were examined in our cohort. Two *FI* measures, a *full FI* based on the 83-item measure developed by Hogan et al. [22] using the approach of Searle and colleagues [21] and a *modified FI* modeled on the original 50-item measure developed by Armstrong et al. [24] were calculated. Both indices were derived from automated and previously collected clinical assessment data (available from the RAI-HC) and were calculated as the proportion of accumulated to potential health deficits. Our methodological and conceptual approach to developing the two *FI* measures was consistent with our previous assisted living study [22]. Due to minor differences across versions of the RAI tools, the adapted *full FI* and *modified FI* included 72 and 48 items respectively (see Additional file 1: Table S1). Items with a missing value were removed from both the numerator and denominator when calculating either frailty index for an individual participant. For both *Frailty Indices*, clients were categorized as ‘robust’ (*FI* values <0.2), ‘pre-frail’ (values between 0.2–0.3), and ‘frail’ (values >0.3) in accordance with previous studies [21, 22, 32].

The *CHESS* scale, a health instability measure embedded within the RAI-HC tool, was used as our final frailty measure. *CHESS* scale values range from 0 to 5, with a score of 0 representing low health instability and a score of 5 representing high health instability. As in previous studies, clients were categorized as ‘robust’ (*CHESS* score of 0), ‘pre-frail’ (*CHESS* score of 1), and ‘frail’ (*CHESS* score of 2 or more) [22].

### Study outcomes

Outcomes of interest were death, LTC placement, inpatient hospitalization, and hospitalization with an alternate level of care (ALC) bed stay [see below for an explanation of this term] in the year following the index date. Mortality data were ascertained from the Registered Persons Database (RPDB), which contains basic demographic information for residents eligible to receive publicly-funded health services in Ontario. Admissions to LTC facilities were determined using the Continuing Care Reporting System Long Term Care (CCRS-LTC) database, which captures admission and RAI assessment information on Ontario LTC residents. The Canadian Institute of Health Information’s Discharge Abstract Database (CIHI-DAD) was used to identify all hospitalizations. The CIHI-DAD contains detailed administrative information that permitted the measurement of hospital visits requiring an ALC stay. ALC patients are those who no longer require hospital services, but cannot be discharged because appropriate care is not available elsewhere [6, 33].

### Baseline characteristics

Using the RPDB, we determined each client’s age, sex, and Rurality Index of Ontario (RIO) score. The RIO score is a continuous measure of remoteness that accounts for community size and travel time to basic and advanced medical services [34], and clients were categorized into Rural (RIO score ≥40), Urban (RIO score 10–39), and Major Urban (RIO score 0–9) areas.

The Ontario Drug Benefit (ODB) database contains prescription medication claims for those covered under the provincial drug program, mainly those aged 65 years and older. We used the ODB database to count the number of unique prescription medications dispensed which overlapped the index date. Hospital and emergency department use in the year prior to index date was enumerated using CIHI-DAD and National Ambulatory Care Reporting System (NACRS), respectively. A general measure of comorbidity, the number of Adjusted Diagnosis Groups (ADGs) categories, was derived using the Johns Hopkins Adjusted Clinical Group Case-Mix System and based on diagnostic information from health services use in the 2 years prior to the index date [35]. We also derived marital status and measures of caregiver availability, support, and distress from assessment items in the RAI-HC tool.

### Statistical analysis

For each frailty measure, we compared the baseline characteristics between ‘robust’, ‘pre-frail’, and ‘frail’ individuals using one-way analysis of variance for continuous variables and chi-square tests for categorical variables. Note, for ease of presentation, descriptive data for the *full FI* is presented in the main manuscript with comparable data for the *modified FI* and *CHESS* provided in the Additional file 1. A weighted Kappa statistic was used to assess the agreement between the *full FI*, the *modified FI,* and the *CHESS* scale using a 3-level risk categorization [36]. All outcomes were treated as binary variables (e.g., occurred or did not occur during the year of follow-up). We examined the unadjusted and adjusted associations between the three frailty measures and our study outcomes, separately, using log-binomial regression models to estimate risk ratios. Adjusted models controlled for age, sex, and general comorbidity measured using ADG groupings. We restricted our covariates to age, sex, and comorbidity to facilitate comparisons with previous research [22] and because of the need to adopt a parsimonious approach to modeling given that other potential covariates are already captured by the FI. For all frailty measures, those defined as ‘robust’ served as the reference category. When examining hospitalizations with an ALC stay, those hospitalized without an ALC stay (*N* = 66,764) were removed from the analysis.

To compare the predictive validity for each frailty measure in relation to our study outcomes, we calculated the area under the receiver operating characteristic curve (AUC) for a model that included age, sex, and comorbidity, and for models that included age, sex, comorbidity, and each frailty measure separately. Using the nonparametric approach proposed by Delong [37], we calculated the p-values comparing the AUC of each respective model to the reference model that included age, sex, comorbidity, and the *full FI*.

All statistical tests were 2-tailed and we defined *P* < 0.05 as the level of statistical significance. Analyses were conducted using SAS Enterprise Guide, Version 6.1 (SAS Institute, Inc.).

## Results

We identified 296,964 long-stay home care clients assessed between April 2010 and March 2013. Clients aged <66 years or >105 years (*N* = 55,367; 18.6 %), those receiving home care exclusively for case management purposes (*N* = 4444; 1.5 %), previous LTC residents (*N* = 2351; 0.8 %), those who died on or prior to cohort entry (*N* = 183; 0.06 %), or clients with a non-Ontario postal code (*N* = 67; 0.02 %) were excluded.

In the cohort analyzed (*N* = 234,552 long-stay home care clients), 19.5 % (95 % Confidence Interval [CI] 19.3–19.7), 24.4 % (95 % CI 24.2–24.6), and 44.1 % (95 % CI 43.9–44.3) were categorized as frail using the *full FI, modified FI,* and *CHESS* scale*,* respectively (Table 1). The prevalence of frailty was 19.3 % (95 % CI 19.1–19.5), 24.0 % (95 % CI 23.8–24.2), and 43.2 % (95 % CI 42.9–43.4) among women and 19.9 % (95 % CI 19.6–20.2), 25.2 % (95 % CI 24.9–25.5), and 45.9 % (95 % CI 45.6–46.2) among men when using the *full FI*, *modified FI,* and *CHESS* scale, respectively (data not shown). The distribution of the continuous versions of the *full FI* and *modified FI* are presented in Additional file 1: Figure S1. During the year following assessment, 17.5 % of long-stay home care clients had died, 17.1 % had been admitted to long-term care homes, 42.0 % had at least one acute care hospitalization, and 13.5 % were hospitalized with an ALC stay.

**Table 1:** Baseline characteristics, frailty status, and study outcomes among long-stay home care recipients in Ontario

Characteristics	Long-stay home care recipients (*N* = 234,552)
*Sociodemographic characteristics*	
Age (years), mean ± SD	82.0 ± 7.42
Sex	
*Male*	83,125 (35.4 %)
*Female*	151,427 (64.6 %)
Rurality Index of Ontario	
*Major Urban*	156,807 (67.2 %)
*Urban*	52,881 (22.7 %)
*Rural*	23,540 (10.1 %)
Marital status	
*Married*	92,064 (39.3 %)
*Never Married/Other*	11,888 (5.1 %)
*Widowed*	115,176 (49.1 %)
*Separated/Divorced*	15,424 (6.6 %)
Primary caregiver	
*No primary caregiver*	5,181 (2.2 %)
*Yes, does not live with client*	110,884 (47.3 %)
*Yes, lives with client*	118,487 (50.5 %)
Caregiver is distressed	54,620 (23.3 %)
Total weekday hours of caregiver support during past week, mean ± SD	11.68 ± 14.06
Total weekend hours of caregiver support during past week, mean ± SD	4.98 ± 5.73
Average hours of caregiver support per day, mean ± SD	2.38 ± 2.78
*Comorbidity*	
Number of ADG comorbidity categories	
*0–5*	32,719 (13.9 %)
*6–9*	71,578 (30.5 %)
*10+*	130,255 (55.5 %)
*Health care utilization*	
Acute care hospitalization in year prior to index date	106,956 (45.6 %)
Acute care hospitalization with ALC stay in year prior to index date	25,870 (11.0 %)
Emergency department visit in year prior to index date	160,770 (68.5 %)
Number of unique prescription medications overlapping index date, mean ± SD	6.05 ± 3.83
Unique prescription medications overlapping index date	
*0–5*	111,120 (47.4 %)
*6–9*	81,204 (34.6 %)
*10+*	42,228 (18.0 %)
*Frailty status*	
Full Frailty Index [FI]	
*Robust*	108,676 (46.3 %)
*Pre-Frail*	80,155 (34.2 %)
*Frail*	45,721 (19.5 %)
Modified Frailty Index [FI]	
*Robust*	95,209 (40.6 %)
*Pre-Frail*	82,107 (35.0 %)
*Frail*	57,236 (24.4 %)
CHESS Scale	
* Robust*	55,241 (23.6 %)
*Pre-Frail*	75,763 (32.3 %)
*Frail*	103,548 (44.1 %)
*Outcomes* ^*a*^ *one year following index date*	
*Death*	41,044 (17.5 %)
*LTC admission*	40,144 (17.1 %)
*Hospitalization*	98,385 (42.0 %)
*Hospitalization with ALC stay*	31,621 (13.5 %)

*ALC* Alternative Level of Care, *CHESS* Changes in Health, End-stage disease and Signs and Symptoms; *LTC* Long-Term Care

a - Outcome categories are not mutually exclusive

Clients categorized as frail on all three measures were older, received more hours of unpaid caregiver support per week, and were more likely to have unpaid caregivers reporting distress (Table 2 for baseline characteristics by the *full FI*; Additional file 1: Tables S2 and S3 for characteristics by the *modified FI* and *CHESS* scale, respectively). Additionally, frail clients had greater comorbidity as measured by the number of ADG categories than pre-frail and robust individuals, were taking more prescription drugs, and had more hospital admissions and emergency department visits in the year prior to the index date.

**Table 2:** Baseline characteristics and outcomes for long-stay home care recipients in Ontario, by the full frailty index

Characteristics^a^	Full Frailty Index [FI]			*P*-value
	Robust (*N* = 108,676)	Pre-Frail (*N* = 80,155)	Frail (*N* = 45,721)	
*Sociodemographic characteristics*				
Age (years), mean ± SD	81.52 ± 7.45	82.11 ± 7.33	82.94 ± 7.42	<.001
Sex				<.001
*Male*	38,967 (35.9 %)	27,606 (34.4 %)	16,552 (36.2 %)	
*Female*	69,709 (64.1 %)	52,549 (65.6 %)	29,169 (63.8 %)	
Rurality Index of Ontario				<.001
*Major Urban*	71,478 (66.1 %)	53,723 (67.4 %)	31,606 (69.6 %)	
*Urban*	25,243 (23.3 %)	17,965 (22.6 %)	9,673 (21.3 %)	
*Rural*	11,406 (10.5 %)	7,974 (10.0 %)	4,160 (9.2 %)	
Marital status				<.001
*Married*	42,380 (39.0 %)	31,121 (38.8 %)	18,563 (40.6 %)	
*Never Married/Other*	6,340 (5.8 %)	3,658 (4.6 %)	1,890 (4.1 %)	
*Widowed*	52,399 (48.2 %)	40,025 (49.9 %)	22,752 (49.8 %)	
*Separated/Divorced*	7,557 (7.0 %)	5,351 (6.7 %)	2,516 (5.5 %)	
Primary caregiver				<.001
*No primary caregiver*	3,133 (2.9 %)	1,441 (1.8 %)	607 (1.3 %)	
*Yes, does not live with client*	54,236 (49.9 %)	37,256 (46.5 %)	19,392 (42.4 %)	
*Yes, lives with client*	51,307 (47.2 %)	41,458 (51.7 %)	25,722 (56.3 %)	
Caregiver is distressed	12,851 (11.8 %)	21,590 (26.9 %)	20,179 (44.1 %)	<.001
Total weekday hours of caregiver support during past week, mean ± SD	8.48 ± 9.67	12.93 ± 13.92	17.10 ± 19.88	<.001
Total weekend hours of caregiver support during past week, mean ± SD	3.72 ± 4.03	5.48 ± 5.67	7.09 ± 8.07	<.001
Average hours of caregiver support per day, mean ± SD	1.74 ± 1.91	2.63 ± 2.74	3.45 ± 3.93	<.001
*Comorbidity*				
Number of ADG comorbidity categories				<.001
*0–5*	16,855 (15.5 %)	10,636 (13.3 %)	5,228 (11.4 %)	
*6–9*	35,508 (32.7 %)	23,905 (29.8 %)	12,165 (26.6 %)	
*10+*	56,313 (51.8 %)	45,614 (56.9 %)	28,328 (62.0 %)	
*Health care utilization*				
Acute care hospitalization in year prior to index date	46,065 (42.4 %)	37,250 (46.5 %)	23,641 (51.7 %)	<.001
Acute care hospitalization with ALC stay in year prior to index date	8,499 (7.8 %)	9,431 (11.8 %)	7,940 (17.4 %)	<.001
Emergency department visit in year prior to index date	68,763 (63.3 %)	56,421 (70.4 %)	35,586 (77.8 %)	<.001
Number of unique prescription medications overlapping index date, mean ± SD	5.52 ± 3.49	6.58 ± 3.89	6.39 ± 4.33	<.001
Unique prescription medications overlapping index date				<.001
*0–5*	58,246 (53.6 %)	33,155 (41.4 %)	19,719 (43.1 %)	
*6–9*	36,256 (33.4 %)	29,605 (36.9 %)	15,343 (33.6 %)	
*10+*	14,174 (13.0 %)	17,395 (21.7 %)	10,659 (23.3 %)	
*Outcomes* ^*b*^ * one year following index date*				
Death	13,212 (12.2 %)	14,307 (17.8 %)	13,525 (29.6 %)	<.001
LTC admission	9,372 (8.6 %)	15,308 (19.1 %)	15,464 (33.8 %)	<.001
Hospitalization	40,850 (37.6 %)	35,876 (44.8 %)	21,659 (47.4 %)	<.001
Hospitalization with ALC stay	11,927 (11.0 %)	12,205 (15.2 %)	7,489 (16.4 %)	<.001

*ADG* Adjusted Diagnosis Groups, *ALC* Alternative Level of Care, *LTC* Long-Term Care, *SD* Standard Deviation

a - Data are presented as N (column %) unless otherwise noted

b - Outcome categories are not mutually exclusive

Between the three frailty measures, the highest level of agreement was between the *full FI* and the *modified FI* (agreement = 77.0 %; weighted kappa = 0.72, 95 % CI 0.72–0.73) (Table 3). There was markedly less agreement between either the *full FI* (weighted kappa = 0.28, 95 % CI 0.28–0.29) or the *modified FI* (weighted kappa = 0.22, 95 % CI 0.22–0.23) with the *CHESS* scale.

**Table 3:** Summary of agreement between frailty measures

Frailty index comparison	Agreement (%)	Weighted Kappa (95 % CI)
Full Frailty Index [FI] - Modified Frailty Index [FI]	77.0	0.72 (0.72–0.73)
Full Frailty Index [FI] – CHESS scale	43.9	0.28 (0.28–0.29)
Modified Frailty Index [FI] – CHESS scale	42.1	0.22 (0.22–0.23)

*CI* Confidence interval

For all three frailty measures, clients defined as pre-frail or frail had a greater risk for all study outcomes (death, LTC admission, hospitalization, and hospitalization with ALC stay) compared with robust individuals after adjustment for age, sex and comorbidity (Table 4). The risk ratios for death and LTC admission were larger than those observed for the hospitalization outcomes. When comparing frail with robust individuals, the *full FI* demonstrated the strongest association with LTC admission (RR = 3.84, 95 % CI 3.75–3.93) while the *CHESS* scale demonstrated the strongest adjusted associations with hospitalization (RR = 1.34, 95 % CI 1.32–1.36), and hospitalization with ALC stay (RR = 1.62, 95 % CI 1.58–1.67).

**Table 4:** Associations between frailty measures and outcomes at one year following index date

Frailty index	Model	Frailty level^a^	Outcomes at one year, risk ratio (95 % CI)			
			Death	LTC admission	Hospitalization	Hospitalization with ALC stay^b^
Full frailty index [FI]	Unadjusted	Pre-Frail	1.47 (1.44, 1.50)	2.21 (2.16, 2.27)	1.19 (1.18, 1.20)	1.44 (1.41, 1.48)
		Frail	2.43 (2.38, 2.49)	3.92 (3.83, 4.01)	1.26 (1.24, 1.28)	1.59 (1.55, 1.63)
	Adjusted^c^	Pre-Frail	1.45 (1.42, 1.48)	2.20 (2.15, 2.26)	1.17 (1.16, 1.19)	1.42 (1.39, 1.45)
		Frail	2.32 (2.27, 2.37)	3.84 (3.75, 3.93)	1.22 (1.20, 1.23)	1.51 (1.47, 1.55)
Modified frailty index [FI]	Unadjusted	Pre-Frail	1.36 (1.33, 1.39)	2.13 (2.08, 2.19)	1.16 (1.15, 1.17)	1.40 (1.37, 1.44)
		Frail	2.26 (2.22, 2.31)	3.64 (3.55, 3.73)	1.25 (1.24, 1.27)	1.57 (1.53, 1.61)
	Adjusted^c^	Pre-Frail	1.35 (1.32, 1.38)	2.11 (2.05, 2.16)	1.15 (1.14, 1.16)	1.38 (1.35, 1.41)
		Frail	2.16 (2.12, 2.21)	3.58 (3.50, 3.67)	1.21 (1.20, 1.23)	1.50 (1.47, 1.54)
CHESS scale	Unadjusted	Pre-Frail	1.30 (1.26, 1.34)	1.39 (1.35, 1.43)	1.17 (1.15, 1.19)	1.30 (1.26, 1.34)
		Frail	2.36 (2.30, 2.43)	1.96 (1.91, 2.02)	1.40 (1.38, 1.41)	1.69 (1.64, 1.74)
	Adjusted^c^	Pre-Frail	1.29 (1.25, 1.33)	1.40 (1.36, 1.44)	1.15 (1.13, 1.17)	1.28 (1.25, 1.32)
		Frail	2.27 (2.21, 2.33)	1.99 (1.94, 2.04)	1.34 (1.32, 1.36)	1.62 (1.58, 1.67)

*ALC* Alternative Level of Care, *CI* Confidence Interval, *ED* Emergency Department

a – Robust individuals serve as the reference category

b – Individuals hospitalized without an ALC stay removed from analysis (*N* = 66,764)

c – Adjusted for age, sex, and number of ADG comorbidity categories

For all study outcomes, models incorporating measures of frailty in addition to age, sex, and comorbidity had greater AUCs compared with models that included only age, sex, and comorbidity (Table 5). The magnitude of the differences was highest when predicting death and LTC admission (and to a lesser degree in predicting hospitalization with an ALC stay) but relatively lower in models predicting hospitalization. For predicting death, the AUCs were highest for the models incorporating the *full FI* and the *CHESS* scale with no significant difference between these two models (*p* = 0.566). The model incorporating the *full FI* was a stronger predictor of LTC admission than models utilizing the other frailty measures. For hospitalization, the models including the *CHESS* scale had significantly higher AUCs, although the incremental gain in AUC compared to models with the other frailty measures was small in absolute terms. The models with the highest AUCs for predicting hospitalization with an ALC stay incorporated either the *full FI* or the *CHESS* scale with no significant difference between the two (*p* = 0.999).

**Table 5:** Comparison of the area under the receiver operating characteristic curves (AUC) for models using different frailty measures

Outcome	Model predictors	AUC (95 % CI)	*P*-Value
Death	Age, sex, and comorbidity	0.604 (0.601, 0.607)	<.001
	Age, sex, comorbidity, and CHESS scale	0.658 (0.655, 0.660)	0.566
	Age, sex, comorbidity, and modified frailty index	0.652 (0.649, 0.655)	<.001
	Age, sex, comorbidity, and full frailty index	0.657 (0.654, 0.660)	Reference
LTC admission	Age, sex, and comorbidity	0.573 (0.570, 0.576)	<.001
	Age, sex, comorbidity, and CHESS scale	0.617 (0.614, 0.620)	<.001
	Age, sex, comorbidity, and modified frailty index	0.688 (0.685, 0.690)	<.001
	Age, sex, comorbidity, and full frailty index	0.698 (0.695, 0.701)	Reference
Hospitalization	Age, sex, and comorbidity	0.588 (0.586, 0.591)	<.001
	Age, sex, comorbidity, and CHESS scale	0.607 (0.605, 0.609)	<.001
	Age, sex, comorbidity, and modified frailty index	0.599 (0.597, 0.601)	0.005
	Age, sex, comorbidity, and full frailty index	0.600 (0.597, 0.602)	Reference
Hospitalization with ALC stay^a^	Age, sex, and comorbidity	0.578 (0.574, 0.581)	<.001
	Age, sex, comorbidity, and CHESS scale	0.602 (0.599, 0.606)	0.999
	Age, sex, comorbidity, and modified frailty index	0.600 (0.596, 0.603)	0.002
	Age, sex, comorbidity, and full frailty index	0.602 (0.599, 0.606)	Reference

*AUC* Area Under the receiver operating characteristic Curve, *ALC* Alternative Level of Care, *CI* Confidence Interval, *ED* Emergency Department

a – Individuals hospitalized without an ALC stay removed from analysis (*N* = 66,764)

## Discussion

Among older long-stay home care clients in Ontario, the estimated prevalence of frailty was dependent on the approach taken. When using the *full FI, modified FI,* and *CHESS* scale, 19.5, 24.4, and 44.1 % were categorized as frail, respectively. The variation in prevalence estimates with measures was not unexpected. The two *FI* measures showed substantial agreement with each other [38] but also differ in the frailty domains assessed, with items reflecting social and cognitive deficits not included in the modified *FI*. The two *FI* measures captured a different subgroup of home care clients than the *CHESS* scale, which categorized approximately twice as many clients as frail and demonstrated relatively poor agreement [38] with both *FI* measures. The *CHESS* scale is derived based on a consideration of 9 RAI items and is primarily reflective of an acute change in health status (e.g., vomiting, dyspnea, decreased food/fluid intake, change is functional status). It was initially validated in an institutionalized sample where it was observed to be significantly associated with mortality over a 3 year period [25]. These differences may underlie the better performance of CHESS in predicting hospitalization (though none of the frailty measures performed very well for this outcome) and the *full FI* in predicting institutionalization.

The distribution of continuous *FI* scores (see Additional file 1: Figure S1) show that it is possible to detect a gradient of frailty within an already generally vulnerable population, providing support for the utility of a *FI* in this population. Such variability suggests it may be possible to target care to frail and pre-frail individuals [9, 10, 39, 40], though further work is needed to assess whether interventions to prevent, slow or reverse frailty in community-dwelling older adults [39, 41] actually lead to significant health benefits for older clients.

The various health outcomes examined were common in our older home care cohort. All three frailty measures were associated with a significantly increased risk of death, LTC admission, hospitalization, and hospitalization with ALC stay, independent of age, sex, and comorbidity. Although not directly comparable, the risk ratios suggest relatively stronger associations for LTC admission and death compared with the hospitalization endpoints. However, the incorporation of frailty into these models resulted in only modest improvements in predictive validity. While there were statistically significant differences *between* frailty measures in their ability to predict certain outcomes, the observed AUC differences did not appear clinically meaningful with the possible exception of the enhanced ability of the *full FI* in predicting LTC admission. As noted above, the *full FI* contains more items relevant to risk of institutionalization, which might explain why it outperformed the *modified FI* and the *CHESS* scale for this outcome. If the calculation is automated and based on previously collected clinical assessment data (e.g., the RAI-HC), the additional items in the *full FI* would not detract from its feasibility in this setting.

Our findings of an increased risk for poor health outcomes among frail older home care clients are consistent with prior studies in other vulnerable populations [13, 22, 24, 26]. This would include the observation of relatively stronger associations for LTC admission and death than hospitalization [22]. Our study expands on this previous work by showing an increased risk of hospitalization with an ALC stay among frail long-stay home care clients. In Canada, the predominant discharge destination following an ALC hospitalization is a LTC facility [42]. In light of the strong association with LTC admissions, it is not surprising that the frailty measures showed relatively stronger associations for this hospitalization outcome.

A limitation noted for many of the previously published risk prediction models for hospitalization among older adults has been their failure to incorporate measures of patients’ functional status [43, 44]. Our findings suggest that the addition of functional items, which one could argue are well captured by the frailty measures examined here, may offer little predictive gain for this outcome. Hospitalization risk may be influenced more by factors such as advance care planning (where preferences of clients and their family are elicited and documented), the availability of primary care and alternatives to hospitalization, and patterns of previous health care utilization.

The roughly equivalent prevalence of frailty among male and female clients is at variance with findings from studies based on general community samples where higher rates have been reported among women [12, 15, 16]. However, findings regarding sex differences have been far less consistent among more impaired populations, also varying with the frailty measure employed [45, 46]. Compared with women, men receiving formal home care services exhibit relatively higher levels of need (e.g., greater cognitive and physical impairment) [6]. Further supporting this observation, Armstrong et al. reported that the frailest in their home care sample were more likely to be men than women [24].

We also observed a significant association between increasing frailty levels and an increase in the average hours of client support by unpaid caregivers and in the likelihood for caregivers reporting distress. These associations highlight the potential clinical utility of frailty measures in older home care clients. The possible moderating effects of caregiver (or other psychosocial) factors on associations between frailty and adverse health outcomes is an area in need of further research [40].

The breadth and scope of the linked databases examined and the comparative analyses across different frailty measures and health outcomes are important strengths of our study. Some limitations should be noted. First, we derived our measures of frailty using assessment items from the RAI-HC at baseline and then followed home care clients forward for one year to examine health outcomes. During follow-up, clients may have improved or accrued additional health deficits and, therefore, may have been less or more frail at the time of outcome assessment. Second, we limited our analyses to frailty measures derived from existing RAI-HC data. We were not able to evaluate other commonly employed frailty definitions (e.g., the Cardiovascular Health Study physical phenotype [12]) in our comparative analyses. Finally, while the RAI-HC is completed by trained professionals using the best available information (e.g., case discussions with physicians and other providers, health record review) and there is support for its reliability and validity overall [31], selected items may be more vulnerable to possible misclassification bias [47, 48] than others.

## Conclusions

The *full FI, modified FI,* and the *CHESS* scale led to different estimates of the prevalence of frailty among long-stay home care clients in Ontario. At the same time, all frailty measures were able to discriminate between clients at lower and higher risk for outcomes such as death and institutionalization. Of the three approaches examined, the *full FI* most accurately predicted LTC admission while the *CHESS* scale was better at predicting hospitalization though none of the measures performed well for this outcome. We showed that it is feasible to calculate a comprehensive *FI* as a derived variable in a population-based home care sample using routinely collected data. This has potential to facilitate research on frailty within this sector and foster greater consideration of the impact of frailty on health care resource planning and evaluation.

Important areas for further work include an examination of the sensitivity to change of different frailty measures and their role in predicting and/or modifying the likelihood of other health outcomes relevant to older home care clients (e.g., falls, functional decline, and drug related adverse events) [49]. Finally, there is an urgent need to develop and evaluate interventions for frailty in this vulnerable population.

## Abbreviations

ADGs, adjusted diagnosis groups; ALC, alternate level of care; AUC, area under the receiver operating characteristic curve; CHESS, changes in health, end-stage disease and signs and symptoms; ED, Emergency Department; FI, frailty index; LTC, long-term care; RAI, resident assessment instrument; RAI-HC, resident assessment instrument – home care

## Additional file

Additional file 1: Table S1. (List of items used to derive full frailty index and modified frailty index); **Table S2.** (Baseline characteristics and outcomes by modified frailty index); **Table S3.** (Baseline characteristics and outcomes by CHESS scale); **Figure S1.** (Distribution of modified and full frailty index). (DOCX 42.2 kb)
