# Supplementary material for: The prevalence and health consequences of frailty in a population-based older home care cohort: a comparison of different measures
Source: BMC Geriatr. 2016 Jul 7;16:133. doi: 10.1186/s12877-016-0309-z (PMC4937594; doi:10.1186/s12877-016-0309-z)
Supplement: Additional file 1: Table S1. — (List of items used to derive full frailty index and modified frailty index); Table S2. (Baseline characteristics and outcomes by modified frailty index); Table S3. (Baseline characteristics and outcomes by CHESS scale); Figure S1. (Distribution of modified and full frailty index). (DOCX 42.2 kb) [file 12877_2016_309_MOESM1_ESM.docx]

**Additional file 1: Appendices**

**Appendix Table 1. List of items in the RAI-HC tool used to derive the full frailty index and the modified frailty index.**

| **RAI-HC item description** | **Full frailty index (72 items)** | **Modified frailty index (48 items)** |
| --- | --- | --- |
| ***Psychosocial well-being*** |  |  |
| Recent decline in level of participation in social activities | ✓ |  |
| Socially isolated during day | ✓ |  |
| Client indicates feelings of loneliness | ✓ |  |
| ***Mood*** |  |  |
| Makes negative statements | ✓ | ✓ |
| Exhibits persistent anger | ✓ | ✓ |
| Expresses unrealistic fears | ✓ | ✓ |
| Repetitive health complaints | ✓ | ✓ |
| Repetitive anxious complaints | ✓ |  |
| Sad, pained, worried facial expressions | ✓ | ✓ |
| Crying, tearfulness | ✓ |  |
| Withdrawal from activities of interest | ✓ | ✓ |
| Reduced social interactions | ✓ | ✓ |
| ***Cognition*** |  |  |
| Impaired cognitive skills for daily decision making | ✓ |  |
| Short-term (5-min recall) memory problem | ✓ |  |
| Procedural memory problem | ✓ |  |
| New onset/worsening of mental function in past 7 days | ✓ |  |
| Change in decision making in past 90 days | ✓ |  |
| ***Communication*** |  |  |
| At least some difficulty to make self understood | ✓ |  |
| At least some difficulty in understanding others | ✓ |  |
| Moderate/severe hearing problems | ✓ |  |
| Moderate/severe vision problems | ✓ | ✓ |
| ***Functional Status and Activity Level*** |  |  |
| At least some difficulty with meal preparation |  | ✓ |
| At least some difficulty with ordinary housework |  | ✓ |
| At least some difficulty with managing finances |  | ✓ |
| At least some difficulty with managing medications |  | ✓ |
| At least some difficulty with phone use | ✓ |  |
| Needs help with stairs | ✓ | ✓ |
| At least some difficulty with shopping | ✓ | ✓ |
| Requires at least some assistance with bathing | ✓ | ✓ |
| Requires at least some assistance with personal hygiene | ✓ | ✓ |
| Requires at least some assistance with dressing upper body | ✓ | ✓ |
| Requires at least some assistance with dressing lower body | ✓ | ✓ |
| Requires at least some assistance with locomotion | ✓ | ✓ |
| Requires at least some assistance with transferring | ✓ | ✓ |
| Requires at least some assistance with toilet use | ✓ | ✓ |
| Requires at least some assistance with bed mobility | ✓ |  |
| Requires at least some assistance with eating | ✓ | ✓ |
| Less than 2 hours of physical activity in past 3 days | ✓ |  |
| Does not leave house at all in typical week | ✓ |  |
| Decline in an Activity of Daily Living in last 90 days | ✓ |  |
| ***Incontinence*** |  |  |
| Some to Daily bladder incontinence | ✓ | ✓ |
| Some to Daily bowel incontinence | ✓ | ✓ |
| ***Disease Diagnoses*** |  |  |
| Hip fracture, other fractures, osteoporosis | ✓ | ✓ |
| Arthritis | ✓ | ✓ |
| Alzheimer disease/Dementia | ✓ | ✓ |
| Head Trauma | ✓ | ✓ |
| Hemiplegia | ✓ | ✓ |
| Multiple sclerosis | ✓ | ✓ |
| Parkinsonism | ✓ | ✓ |
| Stroke or CVA | ✓ | ✓ |
| Hypertension | ✓ | ✓ |
| Coronary artery disease | ✓ | ✓ |
| Congestive heart failure | ✓ | ✓ |
| Emphysema/COPD/asthma | ✓ | ✓ |
| Cancer | ✓ |  |
| Diabetes | ✓ | ✓ |
| Renal failure | ✓ | ✓ |
| Peripheral vascular disease | ✓ | ✓ |
| Irregularly irregular pulse | ✓ | ✓ |
| Thyroid disease | ✓ | ✓ |
| ***Presence of other health conditions*** |  |  |
| At least one fall in last 90 days | ✓ |  |
| Dizziness in past 3 days | ✓ |  |
| Unsteady gait | ✓ | ✓ |
| Chest pain | ✓ |  |
| Delusions | ✓ |  |
| Hallucinations | ✓ |  |
| Vomiting present last 3 days | ✓ |  |
| Edema | ✓ |  |
| Shortness of breath | ✓ |  |
| Pain | ✓ |  |
| Client reports poor self-rated health | ✓ | ✓ |
| Unstable health condition | ✓ | ✓ |
| ***Nutritional status and medications*** |  |  |
| Severe malnutrition | ✓ | ✓ |
| Morbid obesity | ✓ | ✓ |
| Unintended weight loss of 5% or more in past 30 days / 10% or more in past 180 days | ✓ | ✓ |
| Problems chewing |  | ✓ |
| 9 or more medications | ✓ |  |

**Appendix Table 2. Baseline characteristics and outcomes for long-stay home care recipients in Ontario,**

**by the modified frailty index.**

| **Characteristics^a^** | **Modified Frailty Index [FI]** | | | **P-value** |
| --- | --- | --- | --- | --- |
|  | **Robust (N=95,209)** | **Pre-Frail (N=82,107)** | **Frail (N=57,236)** |  |
| *Sociodemographic characteristics* |  |  |  |  |
| Age (years), mean ± SD | 81.42 ± 7.44 | 82.26 ± 7.32 | 82.59 ± 7.46 | <.001 |
| Sex |  |  |  | <.001 |
| *Male* | 34,091 (35.8%) | 28,102 (34.2%) | 20,932 (36.6%) |  |
| *Female* | 61,118 (64.2%) | 54,005 (65.8%) | 36,304 (63.4%) |  |
| Rurality Index of Ontario |  |  |  | <.001 |
| *Major Urban* | 61,661 (65.1%) | 55,043 (67.4%) | 40,103 (70.5%) |  |
| *Urban* | 22,428 (23.7%) | 18,686 (22.9%) | 11,767 (20.7%) |  |
| *Rural* | 10,625 (11.2%) | 7,899 (9.7%) | 5,016 (8.8%) |  |
| Marital status |  |  |  | <.001 |
| *Married* | 34,820 (36.6%) | 32,211 (39.2%) | 25,033 (43.7%) |  |
| *Never Married/Other* | 5,731 (6.0%) | 3,836 (4.7%) | 2,321 (4.1%) |  |
| *Widowed* | 47,416 (49.8%) | 40,753 (49.6%) | 27,007 (47.2%) |  |
| *Separated/Divorced* | 7,242 (7.6%) | 5,307 (6.5%) | 2,875 (5.0%) |  |
| Primary caregiver |  |  |  | <.001 |
| *No primary caregiver* | 3,069 (3.2%) | 1,431 (1.7%) | 681 (1.2%) |  |
| *Yes, does not live with client* | 50,560 (53.1%) | 38,187 (46.5%) | 22,137 (38.7%) |  |
| *Yes, lives with client* | 41,580 (43.7%) | 42,489 (51.7%) | 34,418 (60.1%) |  |
| Caregiver is distressed | 10,999 (11.6%) | 20,493 (25.0%) | 23,128 (40.4%) | <.001 |
| Total weekday hours of caregiver support during past week, mean ± SD | 7.74 ± 8.90 | 12.25 ± 13.09 | 17.42 ± 19.31 | <.001 |
| Total weekend hours of caregiver support during past week, mean ± SD | 3.43 ± 3.75 | 5.20 ± 5.33 | 7.23 ± 7.84 | <.001 |
| Average hours of caregiver support per day, mean ± SD | 1.60 ± 1.76 | 2.49 ± 2.58 | 3.52 ± 3.82 | <.001 |
| *Comorbidity* |  |  |  |  |
| Number of ADG comorbidity categories |  |  |  | <.001 |
| *0-5* | 14,732 (15.5%) | 11,252 (13.7%) | 6,735 (11.8%) |  |
| *6-9* | 31,516 (33.1%) | 24,860 (30.3%) | 15,202 (26.6%) |  |
| *10+* | 48,961 (51.4%) | 45,995 (56.0%) | 35,299 (61.7%) |  |
| *Health care utilization* |  |  |  |  |
| Acute care hospitalization in year prior to index date | 40,130 (42.1%) | 37,329 (45.5%) | 29,497 (51.5%) | <.001 |
| Acute care hospitalization with ALC stay in year prior to index date | 6,778 (7.1%) | 9,235 (11.2%) | 9,857 (17.2%) | <.001 |
| Emergency department visit in year prior to index date | 60,603 (63.7%) | 56,513 (68.8%) | 43,654 (76.3%) | <.001 |
| Number of unique prescription medications overlapping index date, mean ± SD | 5.38 ± 3.44 | 6.52 ± 3.81 | 6.50 ± 4.30 | <.001 |
| Unique prescription medications overlapping index date |  |  |  | <.001 |
| *0-5* | 52,585 (55.2%) | 34,420 (41.9%) | 24,115 (42.1%) |  |
| *6-9* | 31,108 (32.7%) | 30,654 (37.3%) | 19,442 (34.0%) |  |
| *10+* | 11,516 (12.1%) | 17,033 (20.7%) | 13,679 (23.9%) |  |
| *Outcomes^b^ one year following index date* |  |  |  |  |
| Death | 11,607 (12.2%) | 13,635 (16.6%) | 15,802 (27.6%) | <.001 |
| LTC admission | 7,984 (8.4%) | 14,683 (17.9%) | 17,477 (30.5%) | <.001 |
| Hospitalization | 35,710 (37.5%) | 35,744 (43.5%) | 26,931 (47.1%) | <.001 |
| Hospitalization with ALC stay | 10,316 (10.8%) | 12,133 (14.8%) | 9,172 (16.0%) | <.001 |
|  |  |  |  |  |

ADG=Adjusted Diagnosis Groups; ALC=Alternative Level of Care; LTC=Long-Term Care; SD=Standard Deviation;

a - Data are presented as N (column %) unless otherwise noted

b - Outcome categories are not mutually exclusive

**Appendix Table 3. Baseline characteristics and outcomes for long-stay home care recipients in Ontario,**

**by the Changes in Health, End-stage disease and Signs and Symptoms (CHESS) scale.**

| **Characteristics^a^** | **CHESS Scale** | | | **P-value** |
| --- | --- | --- | --- | --- |
|  | **Robust (N=55,241)** | **Pre-Frail (N=75,763)** | **Frail (N=103,548)** |  |
| *Sociodemographic characteristics* |  |  |  |  |
| Age (years), mean ± SD | 81.85 ± 7.56 | 82.02 ± 7.40 | 82.07 ± 7.36 | <.001 |
| Sex |  |  |  | <.001 |
| *Male* | 19,361 (35.0%) | 25,620 (33.8%) | 38,144 (36.8%) |  |
| *Female* | 35,880 (65.0%) | 50,143 (66.2%) | 65,404 (63.2%) |  |
| Rurality Index of Ontario |  |  |  | <.001 |
| *Major Urban* | 37,948 (69.0%) | 51,900 (68.8%) | 66,959 (65.1%) |  |
| *Urban* | 11,730 (21.3%) | 16,406 (21.8%) | 24,745 (24.1%) |  |
| *Rural* | 5,286 (9.6%) | 7,091 (9.4%) | 11,163 (10.9%) |  |
| Marital status |  |  |  | <.001 |
| *Married* | 21,255 (38.5%) | 29,354 (38.7%) | 41,455 (40.0%) |  |
| *Never Married/Other* | 3,286 (5.9%) | 3,961 (5.2%) | 4,641 (4.5%) |  |
| *Widowed* | 26,999 (48.9%) | 37,431 (49.4%) | 50,746 (49.0%) |  |
| *Separated/Divorced* | 3,701 (6.7%) | 5,017 (6.6%) | 6,706 (6.5%) |  |
| Primary caregiver |  |  |  | <.001 |
| *No primary caregiver* | 1,682 (3.0%) | 1,773 (2.3%) | 1,726 (1.7%) |  |
| *Yes, does not live with client* | 26,179 (47.4%) | 36,468 (48.1%) | 48,237 (46.6%) |  |
| *Yes, lives with client* | 27,380 (49.6%) | 37,522 (49.5%) | 53,585 (51.7%) |  |
| Caregiver is distressed | 7,196 (13.0%) | 14,648 (19.3%) | 32,776 (31.7%) | <.001 |
| Total weekday hours of caregiver support during past week, mean ± SD | 9.80 ± 12.11 | 10.80 ± 12.99 | 13.33 ± 15.52 | <.001 |
| Total weekend hours of caregiver support during past week, mean ± SD | 4.27 ± 4.97 | 4.64 ± 5.29 | 5.60 ± 6.33 | <.001 |
| Average hours of caregiver support per day, mean ± SD | 2.01 ± 2.39 | 2.21 ± 2.56 | 2.70 ± 3.07 | <.001 |
| *Comorbidity* |  |  |  |  |
| Number of ADG comorbidity categories |  |  |  | <.001 |
| *0-5* | 10,024 (18.1%) | 10,693 (14.1%) | 12,002 (11.6%) |  |
| *6-9* | 18,177 (32.9%) | 23,449 (31.0%) | 29,952 (28.9%) |  |
| *10+* | 27,040 (48.9%) | 41,621 (54.9%) | 61,594 (59.5%) |  |
| *Health care utilization* |  |  |  |  |
| Acute care hospitalization in year prior to index date | 18,650 (33.8%) | 33,476 (44.2%) | 54,830 (53.0%) | <.001 |
| Acute care hospitalization with ALC stay in year prior to index date | 3,970 (7.2%) | 8,174 (10.8%) | 13,726 (13.3%) | <.001 |
| Emergency department visit in year prior to index date | 31,038 (56.2%) | 51,284 (67.7%) | 78,448 (75.8%) | <.001 |
| Number of unique prescription medications overlapping index date, mean ± SD | 5.57 ± 3.56 | 5.96 ± 3.75 | 6.38 ± 4.00 | <.001 |
| Unique prescription medications overlapping index date |  |  |  | <.001 |
| *0-5* | 29,012 (52.5%) | 36,735 (48.5%) | 45,373 (43.8%) |  |
| *6-9* | 18,728 (33.9%) | 26,155 (34.5%) | 36,321 (35.1%) |  |
| *10+* | 7,501 (13.6%) | 12,873 (17.0%) | 21,854 (21.1%) |  |
| *Outcomes^b^ one year following index date* |  |  |  |  |
| Death | 5,694 (10.3%) | 10,149 (13.4%) | 25,201 (24.3%) | <.001 |
| LTC admission | 6,092 (11.0%) | 11,628 (15.3%) | 22,424 (21.7%) | <.001 |
| Hospitalization | 18,855 (34.1%) | 30,221 (39.9%) | 49,309 (47.6%) | <.001 |
| Hospitalization with ALC stay | 5,713 (10.3%) | 9,771 (12.9%) | 16,137 (15.6%) | <.001 |
|  |  |  |  |  |

ADG=Adjusted Diagnosis Groups; ALC=Alternative Level of Care; LTC=Long-Term Care; SD=Standard Deviation;

a – Data are presented as N (column %) unless otherwise noted

b – Outcome categories are not mutually exclusive

**Appendix Figure 1. Distribution of modified and full frailty index for all long-stay home care recipients in Ontario.**

.

Modified frailty index (Median = 0.223; Interquartile Range = 0.138; Range = 0.000-0.723) shown in grey and Full frailty index (Median = 0.208; Interquartile Range = 0.125; Range =0.000-0.706) shown in black.
